# Independent introductions and nosocomial transmission of *Candida auris* in Saudi Arabia ─ a genomic epidemiological study of an outbreak from a hospital in Riyadh

**DOI:** 10.1128/spectrum.03260-24

**Published:** 2025-02-04

**Authors:** Qingtian Guan, Faisal Alasmari, Chang Li, Sara Mfarrej, Mahmoud Mukahal, Stefan T. Arold, Talal S. AlMutairi, Arnab Pain

**Affiliations:** 1 Bioscience Program, King Abdullah University of Science and Technology (KAUST), Thuwal-Jeddah, Saudi Arabia; 2 King Fahad Medical City (KFMC), Riyadh, Saudi Arabia; 3 Alfaisal University, Riyadh, Saudi Arabia; 4 KAUST Center of Excellence for Smart Health (KCSH), King Abdullah University of Science and Technology (KAUST), Thuwal-Jeddah, Saudi Arabia; 5 King Saud University Medical City, Riyadh, Saudi Arabia; Brown University, Providence, Rhode Island, USA

**Keywords:** *Candida auris*, Saudi Arabia, genomic epidemiology, antifungal resistance, transmission network

## Abstract

*Candida auris*, a pathogenic yeast responsible for global healthcare-associated outbreaks, was involved in a significant outbreak at King Fahad Medical City (KFMC) in Riyadh, affecting 20 patients from August 2018 to May 2019. Our study analyzed 23 *C*. *auris* isolates from these patients, utilizing whole genome sequencing, single-nucleotide polymorphism (SNP) analysis, phylogenetic analysis, and transmission network construction. We identified four phylogeographic clades in Saudi Arabia, with two present among the KFMC isolates. The transmission network predominantly displayed two star-like patterns, indicative of super-spreader events. Resistance to various azoles was common, and one isolate was resistant to 5-flucytosine, linked to specific mutations in the ERG11 and CIT1 genes. High genetic similarity of the isolates suggested a nosocomial origin of the KFMC outbreak. The distribution of phylogeographic clades suggests at least four separate introductions into Saudi Arabia from 2017 to 2019 and two into KFMC, underscoring both direct and nosocomial transmission pathways within the hospital.

**IMPORTANCE:**

*Candida auris* is an emerging multidrug-resistant yeast that poses a significant threat in healthcare settings worldwide. This study is one of the largest genomic investigations of a *Candida auris* outbreak in the Middle East, focusing on a hospital in Riyadh, Saudi Arabia. By analyzing the genomes of isolates from 20 patients, we uncovered multiple independent introductions of *C. auris* into the region, as well as its subsequent spread within a hospital. The findings highlight the complex transmission dynamics and the challenges in controlling this pathogenic yeast in healthcare environments. This research underscores the critical need for robust genomic surveillance and accurate identification methods to prevent and manage *C. auris* outbreaks, which are increasingly linked to high mortality rates and limited treatment options. The insights gained from this study contribute to our understanding of *C. auris* transmission and resistance, offering valuable guidance for public health strategies.

## INTRODUCTION

*Candida auris* is an emerging multidrug-resistant fungus that causes invasive infections and has been reported in healthcare settings worldwide. *C. auris* was first isolated and identified in 2009 in Japan (1), followed by an increasing number of infections reported in over 40 countries worldwide in the past decade (2–9). It is associated with human-to-human transmission and can also be spread from one patient via shared objects in healthcare settings (3).

Unfortunately, treatment options against this invasive and emerging yeast are limited. Several *C. auris* cases have demonstrated its resistance or reduced susceptibility to three major antifungal classes of drugs: azoles, polyenes, and echinocandins (2). The minimum inhibitory concentration (MIC) is the lowest concentration of an antimicrobial that prevents visible growth of a microorganism. *C. auris* has shown elevated minimum inhibitory MIC to triazole antifungal drugs, including voriconazole, posaconazole, itraconazole, and isavuconazole, and almost all of the *C. auris* case reports showed resistance to fluconazole (10–12). Amphotericin B belongs to polyene antifungal drug, and it shows reduced susceptibility against *C. auris* isolates in some studies (2, 11–15), which is toxic to humans and is used as the last resort for anti-fungal treatment. Considering the resistance of azoles and amphotericin B, echinocandins were recommended as a first-line therapy, while elevated MIC to one or more echinocandins has been reported (15–18).

Reliable protocols for identifying *C. auris* now include loop-mediated isothermal amplification (LAMP) (19), matrix-assisted laser desorption ionization time-of-flight mass spectrometry (MALDI-TOF MS) (20), and PCR/qPCR (21, 22) methods. Historically, *C. auris* was frequently misdiagnosed as other *Candida* species by commonly used diagnostic platforms in hospitals, such as VITEK 2, API 20C, and BD Phoenix (23). This misidentification often delayed proper treatment and increased fatalities. However, with the updates to these systems, including the incorporation of *C. auris* into libraries, the accuracy of diagnosis has improved significantly in recent years, reducing the likelihood of misdiagnosis.

Whole genome sequencing (WGS) analysis of 75 global *C. auris* clinical isolates suggests the independent emergence of five populations of *C. auris* in three continents: South Asia, East Asia, Middle East (Iran), South Africa, and South America (2, 24) designated as clades I–V. Recently, an new clade of *C auris*, clade VI, was proposed based on the new reported cases in Singapore and Bangladesh (25, 26). A previous study showed that Saudi Arabian isolates are genetically related to clade I (27). In recent years, multiple foci of *C. auris* infections have been reported in the Middle East, such as from Iran (24), Saudi Arabia (27–29), Kuwait (30), Israel (31), Oman (32), Qatar (33–35), and the United Arab Emirates (36). However, only a limited number of WGS-based genomic epidemiology studies have been conducted so far from this region.

In this study, we analyzed the genomes of 23 *C*. *auris* isolates from 20 patients in 2019, representing one of the largest recent outbreaks in the Middle East at a hospital in Riyadh. Using an SNP-based phylogenomic tracking analysis combined with hospital clinical records data, we provide evidence that there are multiple introductions of *C. auris* to Saudi Arabia that resulted in subsequent local nosocomial transmissions within a hospital setting.

## RESULTS

### Case description and antimicrobial resistance profile

**Fig 1:**
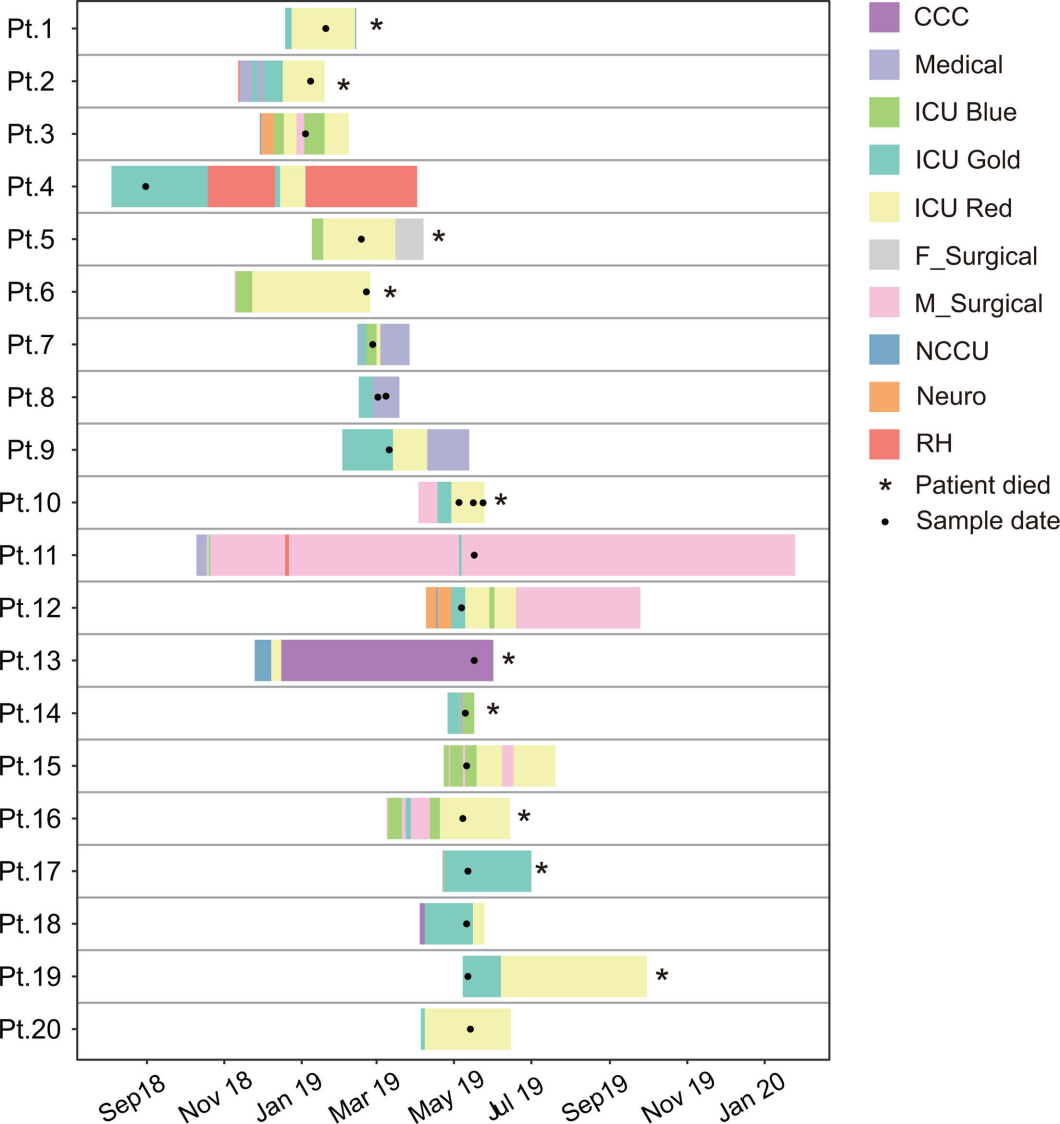
Gantt plot showing the locations of affected patients during their hospital stay before and after acquiring *C. auris*. The *y*-axis has patient IDs, and the *x*-axis represents the date. CCC: comprehensive cancer center ward; Medical: medical ward; ICU bule: ICU bule area ward; ICU gold: ICU gold area ward; ICU red: ICU red area ward; F_Surgical: female surgical ward; M_Surgical: male surgical ward; NCCU: NCCU ward; Neuro: neuro ward; RH: Rehab Hospital. The star symbol indicates the death of the patients after treatment.

Twenty-three samples were derived from twenty patients who were diagnosed with *C. auris* infection between August 2018 to May 2019 at the King Fahad Medica City (KFMC) hospital in Riyadh, Saudi Arabia (Fig. 1, Table 1). Invasive infections had developed in 16 out of 20 patients (80%). The median age was 52 years among the 20 patients; 13 cases were males, while the other seven were females. All cases were misidentified as *Candida* spp. by BD Phoenix and later identified as *C. auris* through ITS sequence amplification and the WGS approach.

**TABLE 1:** *C. auris* isolates and patient information as available in hospital records^*a*^

Patient no.	Age/sex/ nationality	Date of admission	Treatment outcome	Sample ID	Sample collection date	Source	Phoenix 100 ID/AST	18S rDNA identification (by WGS)
Pt.1	Male/53/ Syrian	2018/12/19	Died	CA1	2019/1/20	Blood C&S	*C. haemulonii*	*C. auris*
Pt.2	Female/67/ Saudi Arabia	2018/11/12	Died	CA2	2019/1/8	Pleural Fluid	*Candida spp*	*C. auris*
Pt.3	Male/81/ Saudi Arabia	2018/11/29	Survived	CA3	2019/1/4	Pleural Fluid	*Candida spp*	*C. auris*
Pt.4	Male/21/ Saudi Arabia	2018/8/4	Survived	CA4	2019/8/31	Blood C&S	*C. haemulonii*	*C. auris*
Pt.5	Female/99/ Saudi Arabia	2019/1/9	Died	CA5	2019/2/17	Bile Fluid	*C. haemulonii*	*C. auris*
Pt.6	Male/59/ Saudi Arabia	2018/11/9	Died	CA6	2019/2/21	Urine	*C. haemulonii*	*C. auris*
Pt.7	Female/29/ Saudi Arabia	2019/2/14	Survived	CA7	2019/2/26	Urine	*C. haemulonii*	*C. auris*
Pt.8	Male/65/ Saudi Arabia	2019/2/15	Survived	CA8	2019/3/2	Blood C&S	*C. haemulonii*	*C. auris*
	Male/65/ Saudi Arabia			CA9	2019/3/7	Blood C&S	*C. haemulonii*	*C. auris*
Pt.9	Female/61/ Saudi Arabia	2019/2/2	Survived	CA10	2019/3/11	Urine	*C. haemulonii*	*C. auris*
Pt.10	Male/27/ Saudi Arabia	2019/4/3	Died	CA11	2019/5/5	Urine	*C. haemulonii*	*C. auris*
				CA12	2019/5/20	Urine	*C. haemulonii*	*C. auris*
				CA23	2019/5/25	Urine	*C. haemulonii*	*C. auris*
Pt.11	Male/35/ Saudi Arabia	2018/10/10	Survived	CA13	2019/5/17	Urine	*C. haemulonii*	*C. auris*
Pt.12	Male/59/ Saudi Arabia	2019/4/9	Survived	CA14	2019/5/7	Urine	*C. haemulonii*	*C. auris*
Pt.13	Female/22/ Saudi Arabia	2019/5/16	Died	CA15	2019/5/17	Blood C&S	*C. haemulonii*	*C. auris*
Pt.14	Male/73/ Saudi Arabia	2019/4/26	Died	CA16	2019/5/10	Urine	*C. haemulonii*	*C. auris*
Pt.15	Male/35/ Saudi Arabia	2019/4/23	Survived	CA17	2019/5/11	Groin and axilla	*C. haemulonii*	*C. auris*
Pt.16	Male/81/ Saudi Arabia	2019/3/9	Died	CA18	2019/5/8	Urine	*C. haemulonii*	*C. auris*
Pt.17	Male/62/ Saudi Arabia	2019/4/22	Died	CA19	2019/5/12	Groin and axilla	*C. haemulonii*	*C. auris*
Pt.18	Female/41/ Saudi Arabia	2019/4/4	Died	CA20	2019/5/11	Urine	*C. haemulonii*	*C. auris*
Pt.19	Female/20/ Saudi Arabia	2019/5/8	Survived	CA21	2019/5/12	Groin and axilla	*C. haemulonii*	*C. auris*
Pt. 20	Male/49/ Saudi Arabia	2019/4/5	Died	CA22	2019/5/14	Skin swab	*Candida spp*	*C. auris*

^*a*^ Abbreviations: AMB, amphotericin B; ANI, anidulafungin; CAS, caspofungin; FLC, fluconazole; ITR, itraconazole; 5-FC, 5-flucytosine; MIF, micafungin; POS, posaconazole; VRC, voriconazole; Blood C&S: Blood culture; N/T: Not tested.

All of the strains (100%) were resistant to fluconazole with minimal inhibitory concentration (MIC) ≥64 mg/L; Four strains (17.39%) were resistant to itraconazole; three strains (13.04%) were resistant to posaconazole, and 22 strains (95.65%) were resistant to voriconazole. One strain (4.35%) was resistant to 5-flucytosine. No echinocandin and amphotericin B resistance was recorded.

In our analysis, we observed the discrepancy between the *C. auris* genomic subtype and antimicrobial resistance (AMR) phenotypes from the microbiology laboratory records. For example, the genomic subtype of strains CA1 and CA4 is identical (Fig. S1), yet their recorded AMR profiles differ. The CA4 strain shows an elevated MIC to voriconazole when compared with CA1 (Table 2). The same discrepancy was also observed with CA3 genomic subtype (CA3, 6, 7, 19, and 20) and the CA9 genomic subtype (CA9, 12, 18, and 23).

**TABLE 2:** Susceptibility to nine antifungal agents (mg/L) as available in hospital records^*a*^^,^^*b*^

Patient no.	AMB	ANI	CAS	MIF	FLC	ITR	5-FC	POS	VRC	ERG11	FCY1	CIT1
Pt. 1	1.00	0.5	0.25 S	0.25 S	**>256**	N/T	N/T	N/T	**0.5**	K143R	S79R	
Pt. 2	1.00	0.25	0.125	0.25 S	**>256**	0.25	0.12	0.12	**0.5**	K143R	S79R	
Pt. 3	1.00	0.25	0.25	0.125 S	**>256**	**0.5**	0.12	0.12	**1.00**	K143R	S79R	
Pt. 4	1.00	0.5	0.25	0.25 S	**>256**	N/T	N/T	N/T	**1.00**	K143R	S79R	
Pt. 5	1.00	0.5	0.25	0.25 S	**>256**	N/T	**1.00**	N/T	**1.00**	K143R	S79R	V213A
Pt. 6	1.00	0.25	0.25	0.25 S	**>256**	**0.5**	0.25	**0.25**	**0.5**	K143R	S79R	
Pt. 7	1.00	0.25	0.25	0.125 S	**>256**	N/T	N/T	N/T	**0.5**	K143R	S79R	
Pt. 8	1.00	0.25	0.25	0.125 S	**>256**	N/T	N/T	N/T	**1.00**	K143R	S79R	
	1.00	0.25	0.25	0.25 S	**>256**	N/T	N/T	N/T	0.5	K143R	S79R	
Pt. 9	1.00	0.25	0.25	0.25 S	**>256**	N/T	N/T	N/T	**1.00**	K143R	S79R	
Pt. 10	0.5	0.25	0.25	0.12 S	**256**	0.25	<=0.06	0.12	**0.5**	K143R	S79R	
	0.5	0.25	0.25	0.12 S	**256**	0.25	<=0.06	0.12	**0.5**	K143R	S79R	
	0.5	0.25	0.25	0.12 S	**256**	0.25	<=0.06	0.12	**0.5**	K143R	S79R	
Pt. 11	1.00	0.25	0.25	0.12 S	**256**	**0.5**	0.12	**0.25**	**1.00**	K143R	S79R	
Pt. 12	0.5	0.12	0.12	0.12 S	**64**	0.25	0.12	0.03	**0.12**	K143R	S79R	
Pt. 13	0.5	0.12	0.12	0.12 S	**256**	0.25	<=0.06	0.12	**0.5**	K143R	S79R	
Pt. 14	0.5	0.12	0.12	0.12 S	**256**	0.12	<=0.06	0.06	**0.5**	K143R	S79R	
Pt. 15	0.5	0.12	0.12	0.06 S	**64**	0.03	<=0.06	0.016	0.06	K143R	S79R	
Pt. 16	0.5	0.12	0.12	0.12 S	**256**	0.25	<=0.06	0.06	**0.5**	K143R	S79R	
Pt. 17	0.5	0.12	0.12	0.12 S	**256**	0.25	<=0.06	0.12	**0.5**	K143R	S79R	
Pt. 18	0.5	0.12	0.25	0.12 S	**256**	0.25	<=0.06	0.12	**0.5**	K143R	S79R	
Pt. 19	0.5	0.25	0.25	0.12 S	**256**	0.25	0.12	0.12	**0.5**	K143R	S79R	
Pt. 20	0.5	0.25	0.12	0.12 S	**256**	**0.5**	<=0.06	**0.25**	**1.00**	K143R	S79R	

^*a*^ Abbreviations: AMB, amphotericin B; ANI, anidulafungin; CAS, caspofungin; FLC, fluconazole; ITR, itraconazole; 5-FC, 5-flucytosine; MIF, micafungin; POS, posaconazole; VRC, voriconazole; Blood C&S: Blood culture; N/T: Not tested.

^*b*^ The bold values represent MICs that exceed the breakpoints defined by the US CDC or those reported in the literature.

### Phylogenomics and transmission analysis

**Fig 2:**
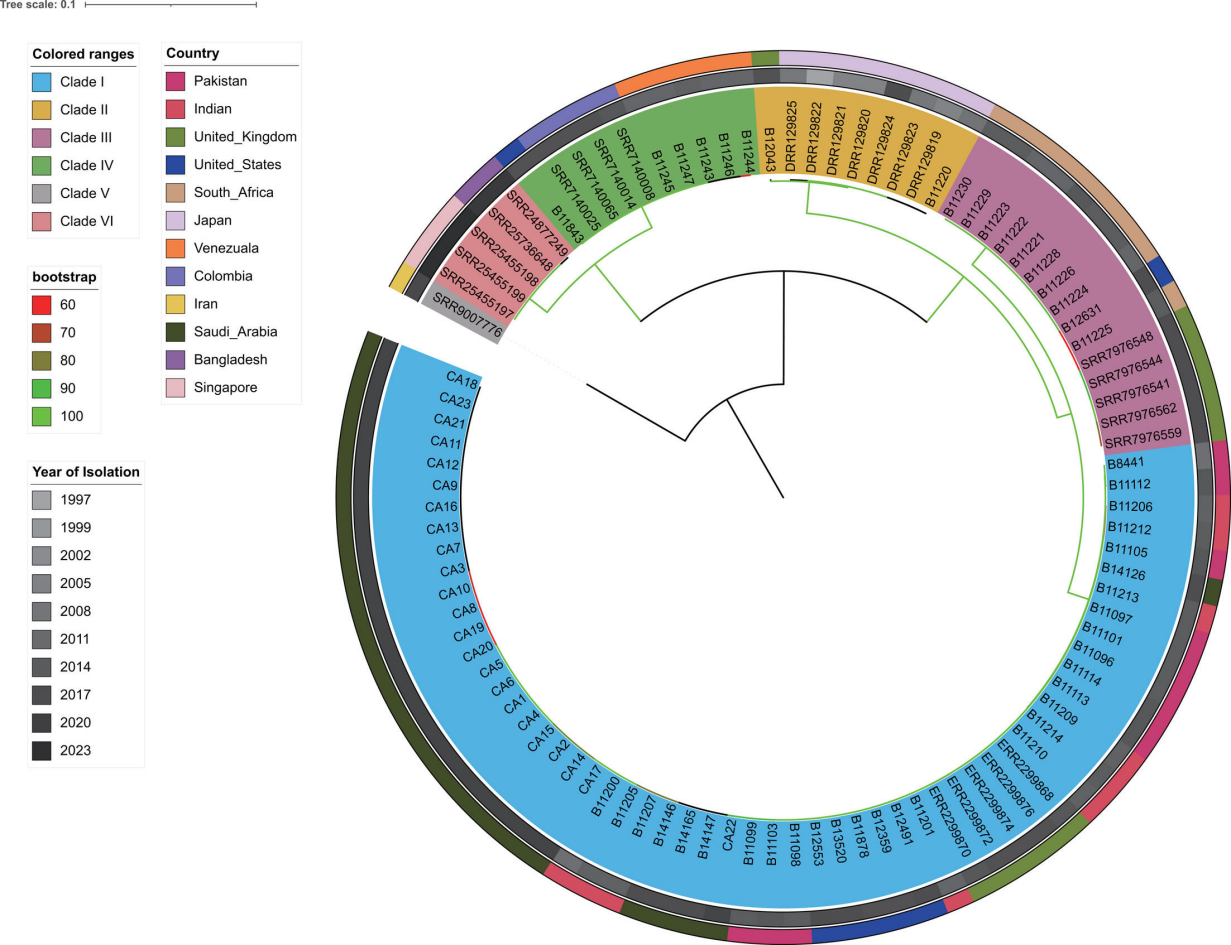
Global phylogeographic comparative analysis of the *C. auris* genomes sampled from KFMC. Maximum-likelihood phylogenetic tree based on 398,899 SNPs shared by 98 strains (genomic data obtained from ENA) across *C. auris* six clades from different countries. The countries, clade information, and bootstrap values are indicated by the legend on the left.

**Fig 3:**
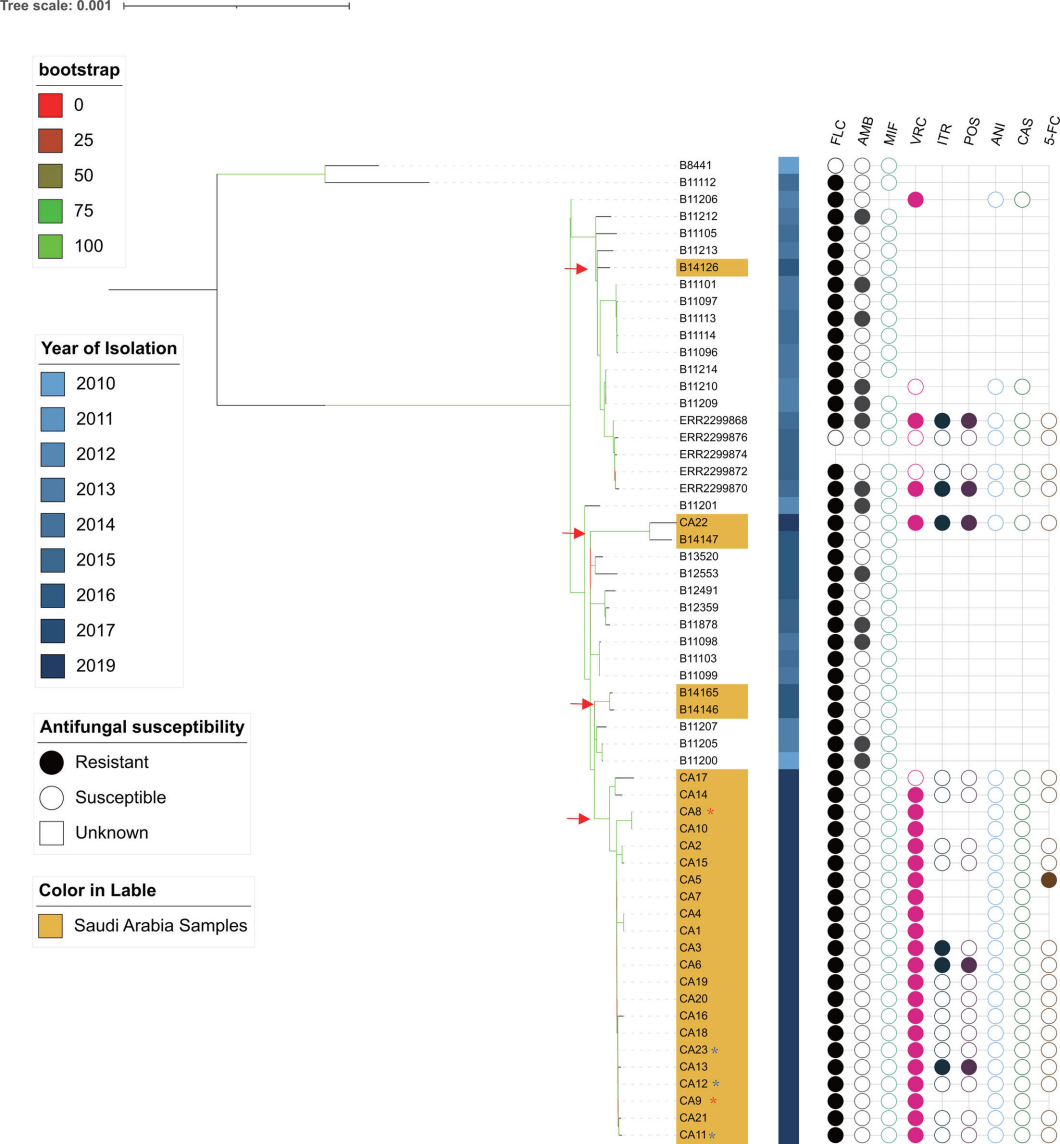
Maximum-likelihood phylogenetic tree of the 58 clade I *C. auris*. A phylogenetic tree was based on SNP information and rooted in clade II B11221 subgroup as the outgroup (due to visualization constraints, the clade II outgroup was omitted from the displayed tree to maintain clarity, the complete phylogenetic tree is shown in Fig. S2). The Saudi Arabian strains were labeled with yellow colors, and four independent introduction events (2017 and 2019) were indicated by red triangles. The samples derived from the same patients were indicated by the same color of star symbol, the bootstrap values by the legend on the left. Information of the year of isolation was indicated by the legend on the left. The antifungal susceptibility profile is displayed on the right panel, showing the resistance (colorful circles), susceptibility (empty circles), or unknown status of each isolate to various antifungal drugs, including fluconazole (FLC), amphotericin B (AMB), voriconazole (VRC), itraconazole (ITR), posaconazole (POS), anidulafungin (ANI), caspofungin (CAS), and 5-flucytosine (5-FC).

*C. auris* isolates that were obtained from KFMC, Riyadh, during the August 2018–May 2019 outbreak were clustered with clinical isolates that belong to clade I (Fig. 2). Four different subgroups of *C. auris* from Saudi Arabia have been identified (Fig. 3), indicating multiple independent introductions and subsequent transmission of *C. auris* in Saudi Arabia. Accordingly, 22 out of 23 isolates (except CA22) formed a tight phylogroup whose pairwise average genetic variation between any two isolates was 17 SNPs, while this population was separated from the other global isolates in clade I by an average of 152 SNPs (Fig. 3; Fig. S2).

**Fig 4:**
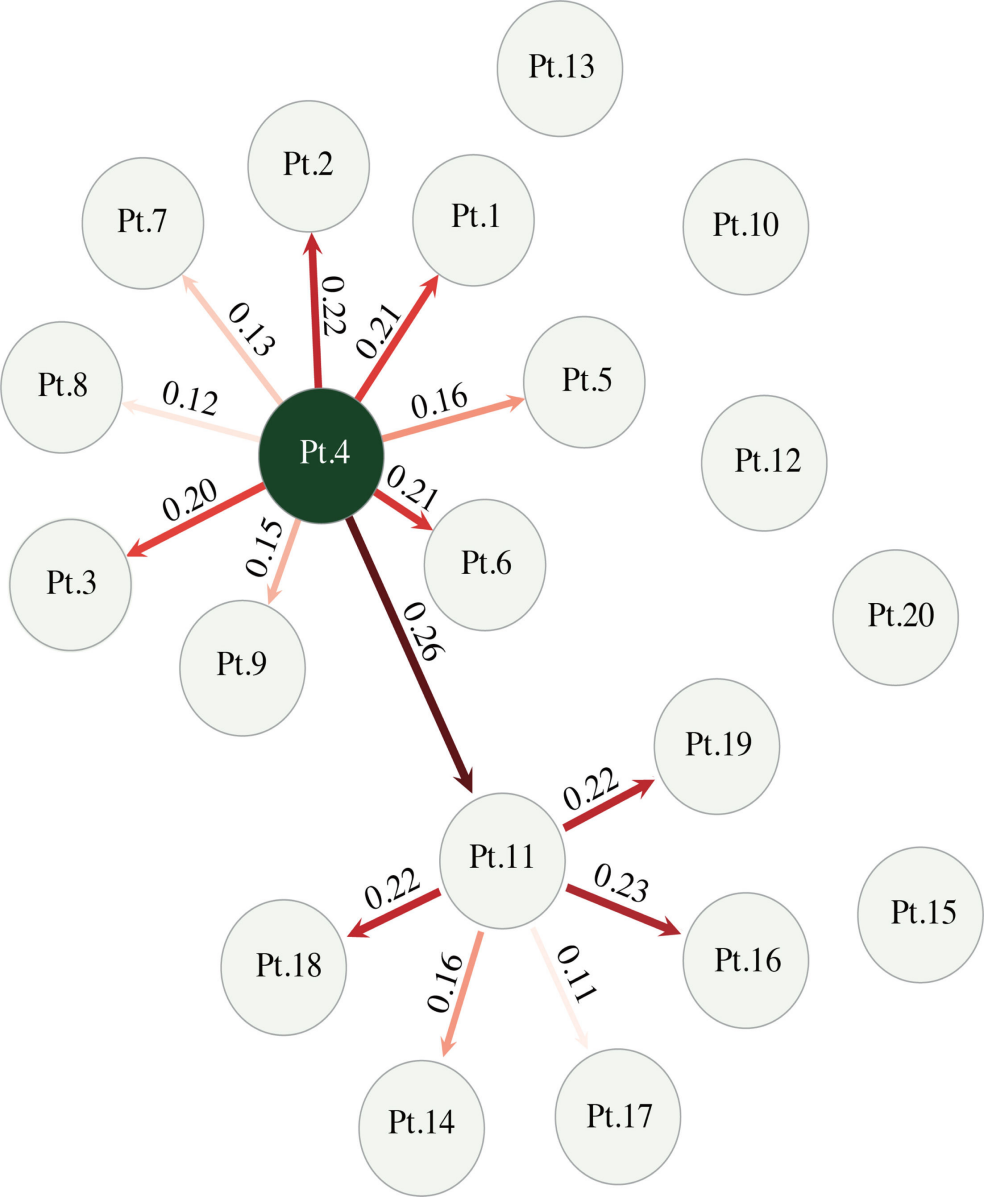
The direct transmission network of the 20 patients infected with *C. auris* in KFMC. Each circle represents one patient. The arrows indicate the deduced transmission direction, and the numbers on the arrows represent the inferred posterior probabilities of the corresponding direct transmission events, and the intensity of the arrow color is proportional to posterior probability.

The direct transmission analysis (Fig. 4) provides the deduced transmission map along with epidemiological data of the 23 samples isolated from 20 patients. The majority of the isolates from patients formed two star-like structures in the transmission map, which supports the presence of super-spreaders (Patients 4 and 11) (Fig. 4). The minimal spanning tree (MST) analysis (Fig. S1) provides the genetic relatedness of the 23 strains. The clustering analysis agrees with the phylogenetic analysis that at least two independent introductions of *C. auris* occurred in the hospital.

**Fig 5:**
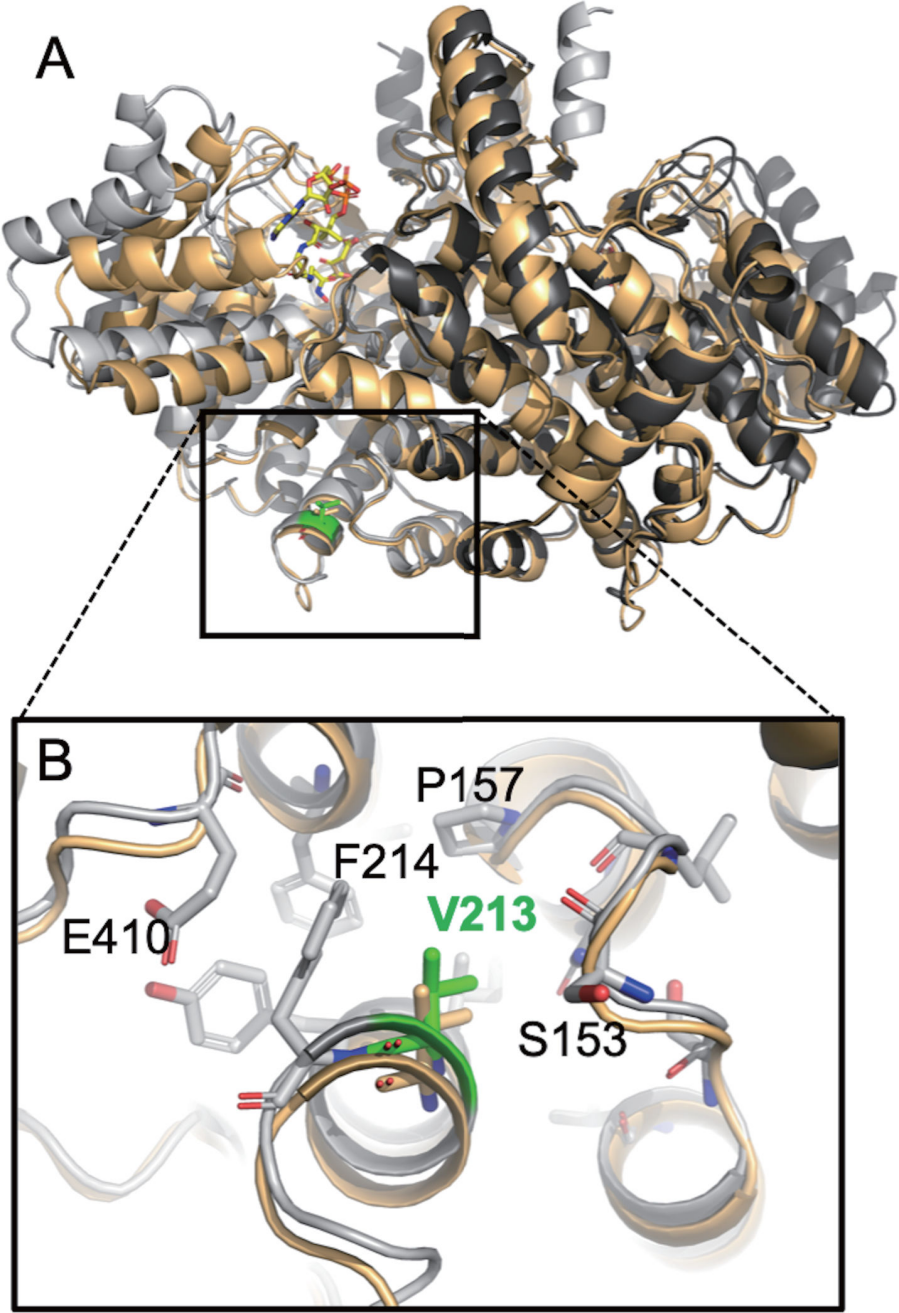
Homology models of *C. auris* CIT1. (**A**) Homology model built based on the open conformation (PDB 5uqs; chains are colored in light and dark gray) superimposed on a model built based on the closed ligand-bound conformation (PDB 1al6; pale orange; bound N-hydroxyamidon-CoA is shown as stick model with carbon atoms in yellow). V213 is shown as a green stick model. (**B**) Zoom into the region of V213. Relevant side chains of the open conformation (gray) are shown. The position of V213 in the closed conformation (pale orange) is shown as stick model. The movement of the Calpha atom of V213 between open and closed conformation is 1.1 Å. The substitution of V213 with an alanine would remove the two gamma carbons on the side chain, leaving a significant gap.

Amino acids substitutions in particular genes are linked to drug resistance of *C. auris* (37). *ERG11* is involved in the ergosterol synthesis pathway and serves as the target for azoles (38). All of the 23 isolates in this study have the K143R mutations in the *ERG11* gene, which agrees with the azole resistance phenotype of the isolates. *FUR1, FCY1*, and *FCY2* genes are involved in the absorption and metabolism of 5-flucytosine, hence, related to its resistance (37). The CA5 strain has an elevated MIC 1.00 mg/L of 5-flucytosine, which is considered resistant (31). We found the *FCY1* S70R mutation in all strains, which is unlikely linked to flucytosine resistance, and no missense mutations were detected in *FUR1* or *FCY2*. Intriguingly, the *CIT1* gene product CIT1, a 461-residue ATP citrate synthase, has a V213A mutation, and it is uniquely present in the CA5 and might be linked to the 5-flucytosine resistance in CA5 as has been previously implicated in studies with laboratory yeast *Saccharomyces cerevisiae (*39*). C. auris* CIT1 is approximately 60% identical in sequence to its orthologs from animals and fungi for which experimental protein structures have been determined and are available in the Protein Data Bank (PDB). Structural modeling of *C. auris* CIT1 models confirmed its dimeric structure and showed that V213A is located more than 20 Å away from the substrate binding site (Fig. 5A). In this position V213A is unable to directly affect the catalytic activity of CIT1. V213 is positioned at the C-terminal end of an alpha helix, where it helps sealing this part of the protein hydrophobic core. The V213A mutation would introduce a gap in this position that may destabilize the protein (Fig. 5B). Comparison of ligand-bound and apo CIT1 structures suggested that this region is also contributing to conformational protein opening and closing required for substrate processing (Fig. 5B). Hence, it is likely that the V213A mutation subtly impairs the catalytic activity of CIT1, however, without completely disrupting its function.

## DISCUSSION

In this study, we presented one of the largest WGS-guided analysis of a *C. auris* outbreak in a hospital setting in the Middle East to date. We confirmed the Saudi Arabian *C. auris* isolates represent four phylotypes, which all belong to South Asian clade (clade I). The high degree of similarity among the isolates in the study population supports the nosocomial transmission of *C. auris* outbreak in KFMC, and the four phylotypes indicate at least four independent introduction events (2017 and 2019) that occurred in Saudi Arabia.

*C. auris* is difficult to identify with standard laboratory tools, and this can lead to inappropriate treatment and delayed infection control precautions. The misidentification of *C. auris* prior to 2019 highlights the limitations of automated identification systems before their libraries were updated. Our retrospective analysis using PCR and WGS played a pivotal role in uncovering the outbreak’s full extent. While this issue has since been addressed with library updates, it underscores the importance of continued molecular surveillance and the re-evaluation of archived samples in similar scenarios. Reliable detection methods include MALDI-TOF MS and PCR/qPCR sequencing. In this study, we demonstrate the advantages of using WGS for identification and transmission tracking (Table 1).

Our current knowledge of *C. auris* resistance is primarily based on the extended knowledge of other *Candida* species, such as *Candida albicans*, while the mechanisms of drug resistance in *C. auris* are largely unknown. In our study, we observed the discrepancy of genomic subtype and phenotypes in terms of MIC of several tested drugs in the sampled *C. auris* strains. Similar results were observed in a study of 304 *C*. *auris* strains by Nancy A. Chow (27), showing that isolates with the same genomic subtype might have different AMR profiles. While this calls for the use of standard optimized protocols for determining MIC values across the clinical microbiology labs, this also highlights the possibility that there might be transcriptional or post-transcriptional regulatory mechanisms related to drug resistance in *C. auris* that is worth further investigations.

The transmission analysis has provided insights into the hypothetical transmission of *C. auris* outbreak in KFMC. It is likely that there is a direct transmission between the first and second clusters (Fig. 4, Patients 4 and 11), and then these super-spreaders caused the subsequently nosocomial transmission. Besides the directly linked related patients, the sporadic cases (Patients 10, 12, 13, 15, and 20) suggest the possibility of unsampled cases or other sources of contagion in the hospital. We have also observed *C. auris* genomic diversity within the same patient: strain CA8 and CA9 isolated from Pt. 8 are separated by 23 SNPs (Fig. S1), which is greater than the average SNP variation, and CA8 and CA9 belong to two different clusters (CA3 and CA9 clusters). It is likely that Pt. 8 has been infected by two independent sources given the fact that the collection date of the two samples (CA8 and CA9) are within a short period of time. The strain CA12 and CA23 have identical genomic background isolated from Pt.10, while CA11 is separated by four SNPs within the same host.

The CA5 strain has an elevated MIC of 1.00 mg/L to 5-flucytosine, and we found the CIT1 V213A mutation uniquely present in CA5, which differs from the CA3, CA6, CA7, CA19, and CA20 isolates by only one SNP. *CIT1* is an ATP citrate synthase, and it is highly induced in biofilms (40) or upon antifungal treatment (39). A study in *Saccharomyces cerevisiae* showed that the antifungal treatments activate mitochondrial activity and shift the cell from fermentation to respiration. This process will lead to overproduction of ROS and result in cell death. Deletion of *CIT1* dramatically reduced yeast sensitivity to three classes of antifungal drugs: amphotericin, miconazole, and ciclopirox (39). The role of CIT1 in *C. auris* drug resistance warrants further exploration, and our hypothesis may shed light on new mechanisms of 5-flucytosine resistance in *C. auris*.

Our findings not only highlight the complex nature of *C. auris* transmission within healthcare settings but also underscore the necessity of continued genomic surveillance to prevent future outbreaks.

## MATERIALS AND METHODS

### Isolates, DNA isolation, taxonomic identification, and susceptibility test

Twenty-three isolates from 20 patients from 10 October 2018 to 16 May 2019 were collected from King Fahad Medical City. The isolates were derived from blood (*n* = 5), pleural fluid (*n* = 2), bile fluid (*n* = 1), urine (*n* = 11), groin and axilla (*n* = 3), and skin (*n* = 1).

At the time of the initial identification (2018–2019), the Phoenix 100 ID/AST system (Becton Dickinson Co, Sparks, Md.) library did not yet include updated entries for *C. auris*, which likely contributed to the misidentification of *C. auris* as *C. haemulonii* or other *Candida* species. To address this, archived *Candida* isolates were retrospectively re-evaluated using PCR-based molecular identification and whole genome sequencing (WGS). Briefly, all the isolates were subjected to DNA extraction using QlAamp DNA Mini Kit (QIAGEN, Germany) followed by PCR using panfungal primers as previously described (41). The obtained amplicons were purified (ExoSAP-IT, Applied Biosystems, Thermo Fisher Scientific, USA) and sequenced using BigDye Terminator v3.1 Cycle Sequencing Kit (Thermo Fisher Scientific, USA) and Genetic Analyzer 3500 (Thermo Fisher Scientific, USA). Susceptibility tests were performed using the microdilution method with Sensititre YeastOne (TREK Diagnostic Systems, Cleveland, Ohio). The breakpoints for *C. auris* are not well-defined; therefore, tentative breakpoints were used according to the US CDC (fluconazole ≥32 mg/L; amphotericin B ≥2 mg/L; anidulafungin ≥4 mg/L; caspofungin ≥2 mg/L; micafungin ≥2 mg/L) and literature (5-flucytosine ≥0.5mg/L; posaconazole ≥0.25 mg/L; voriconazole ≥0.5 mg/L; itraconazole ≥0.5mg/L)(31).

### Sequencing and single-nucleotide polymorphism identification

The genomic DNA from the isolates was then subjected to library preparation using NEBNext (New England Biolabs, USA) and sequenced by Illumina HiSeq 4000 platform with 150 bp paired-end module. The raw reads were first filtered with BBDuk (http://jgi.doe.gov/data-and-tools/bb-tools/) to remove Illumina adapters, phiX, and low-quality bases from both ends to Q20. BWA and GATK were used within the SNP analysis pipeline NASP (1, 42) using B8441 (2) as the reference genome; positions that had <10× coverage or <90% variant allele calls, or within the duplicated regions in the reference were filtered. Phylogenetic analysis was conducted using IQ-TREE (43) with 1,000 bootstrap replicates and the ModelFinder Plus module, which identified TVM+F as the best fitting model for phylogenetic construction. The resulting tree was visualized using iTOL (44). B11221 from clade II was used as the outgroup for the clade I phylogenetic analysis. The minimal spanning tree analysis was performed with PHYLOViZ (45) using the concatenated SNPs obtained from NASP pipeline. The pairwise comparison of the number of SNPs was calculated by VCFtools (46).

### Construction of the transmission network

The Structured COalescent Transmission Tree Inference (SCOTTI, version 1.1.1) package was used to construct the transmission network of patients with default parameters (47). The earliest and latest dates when hosts were infectious and sampling dates were collected for the MCC tree calculation using BEAST2 with default parameters (48).

### *In silico* molecular modeling

Swiss Model (49) was used to produce homology models of *C. auris* CIT1. The open CIT1 conformation was modelled based on the chicken mitochondrial citrate synthase (PDB 5uqs; 59.7% sequence identity, QMEANDisCo score was 0.82 ± 0.05). The ligand-bound closed conformation was modeled based on the same molecule bound to N-hydroxyamidon-CoA and oxaloacetate (PDB 1al6, 61.2% sequence identity, QMEANDisCo score was 0.82 ± 0.05). Proteins were visualized using pymol (pymol.org).

## Data Availability

The raw sequencing data for *Candida auris* isolates are available at European Nucleotide Archive (ENA) under study accession no. PRJEB84203.

## SUPPLEMENTAL MATERIAL

Figures S1 and S2, and File S1 spectrum.03260-24-s0001.pdf Figure S1: Minimum spanning tree of the clustering of 23 isolates from KFMC. Figure S2: Maximum-likelihood phylogenetic tree of the 58 clade I *C. auris*. File S1. Evolutionary tree for Fig. S2 in Newick format, generated using IQ-TREE with 1000 bootstrap replicates.

ASM does not own the copyrights to Supplemental Material that may be linked to, or accessed through, an article. The authors have granted ASM a non-exclusive, world-wide license to publish the Supplemental Material files. Please contact the corresponding author directly for reuse.
