# Supplementary material for: Independent introductions and nosocomial transmission of Candida auris in Saudi Arabia ─ a genomic epidemiological study of an outbreak from a hospital in Riyadh
Source: Microbiol Spectr. 2025 Feb 4;13(3):e03260-24. doi: 10.1128/spectrum.03260-24 (PMC11878057; doi:10.1128/spectrum.03260-24)
Supplement: Figures S1 and S2, and File S1 — Figure S1: Minimum spanning tree of the clustering of 23 isolates from KFMC. Figure S2: Maximum-likelihood phylogenetic tree of the 58 clade I C. auris. File S1. Evolutionary tree for Fig. S2 in Newick format, generated using IQ-TREE with 1000 bootstrap replicates. [file spectrum.03260-24-s0001.pdf]

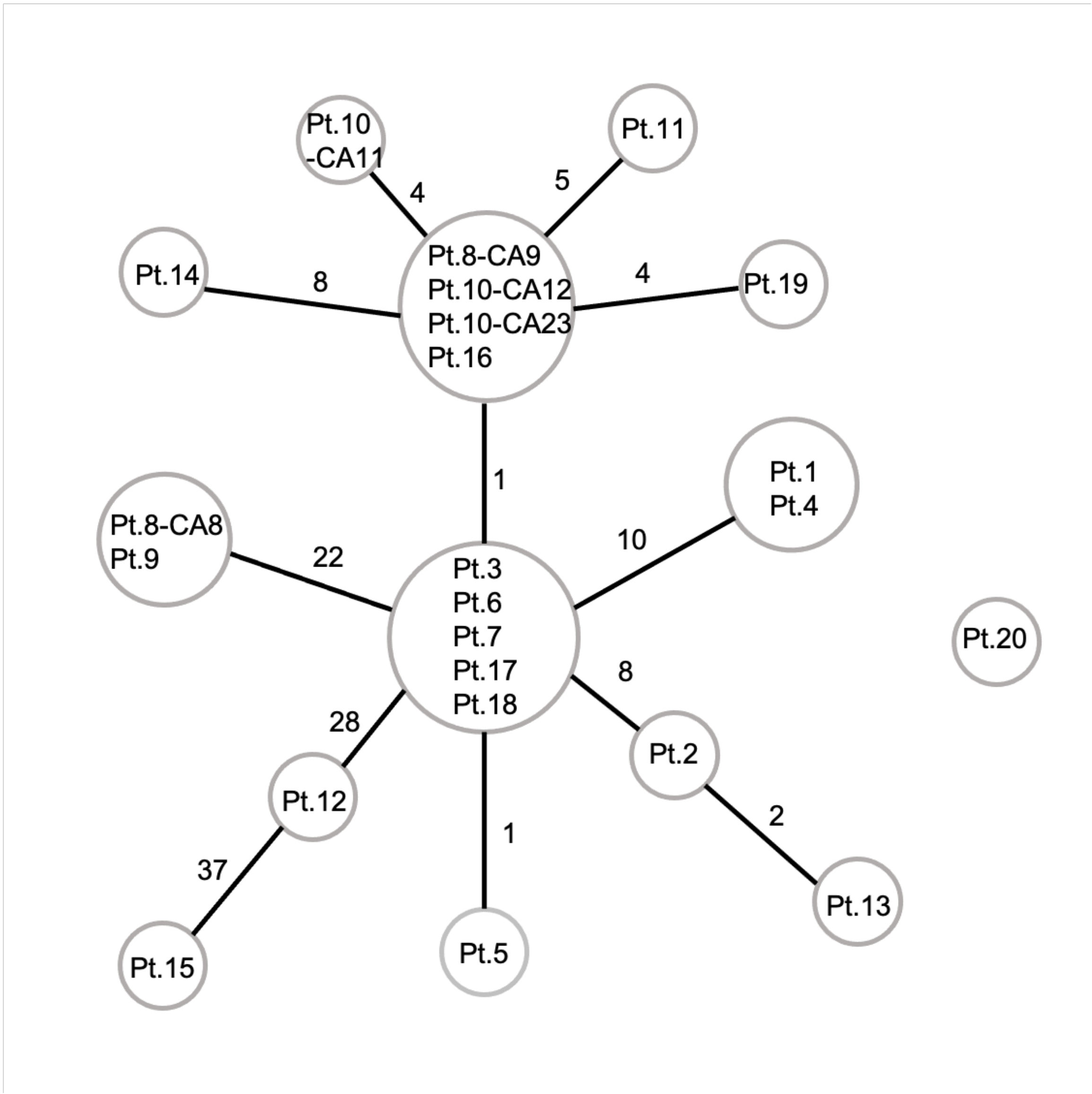

Supplementary Figure 1. Minimum spanning tree of the clustering of 23 isolates from KFMC. Minimum spanning tree analysis with PHYLOViZ [1] based upon concatenated 272 SNPs shared across 23 strains isolated from the 20 patients in this study obtained from NASP pipeline [2], showing the genetical relatedness of the strains isolated from the patients. The number of SNPs separating every 2 close-related isolates is indicated on each branch. The strain number is shown when multiple samples have been collected from the same patient. For details of the isolates, please refer to Table.1.

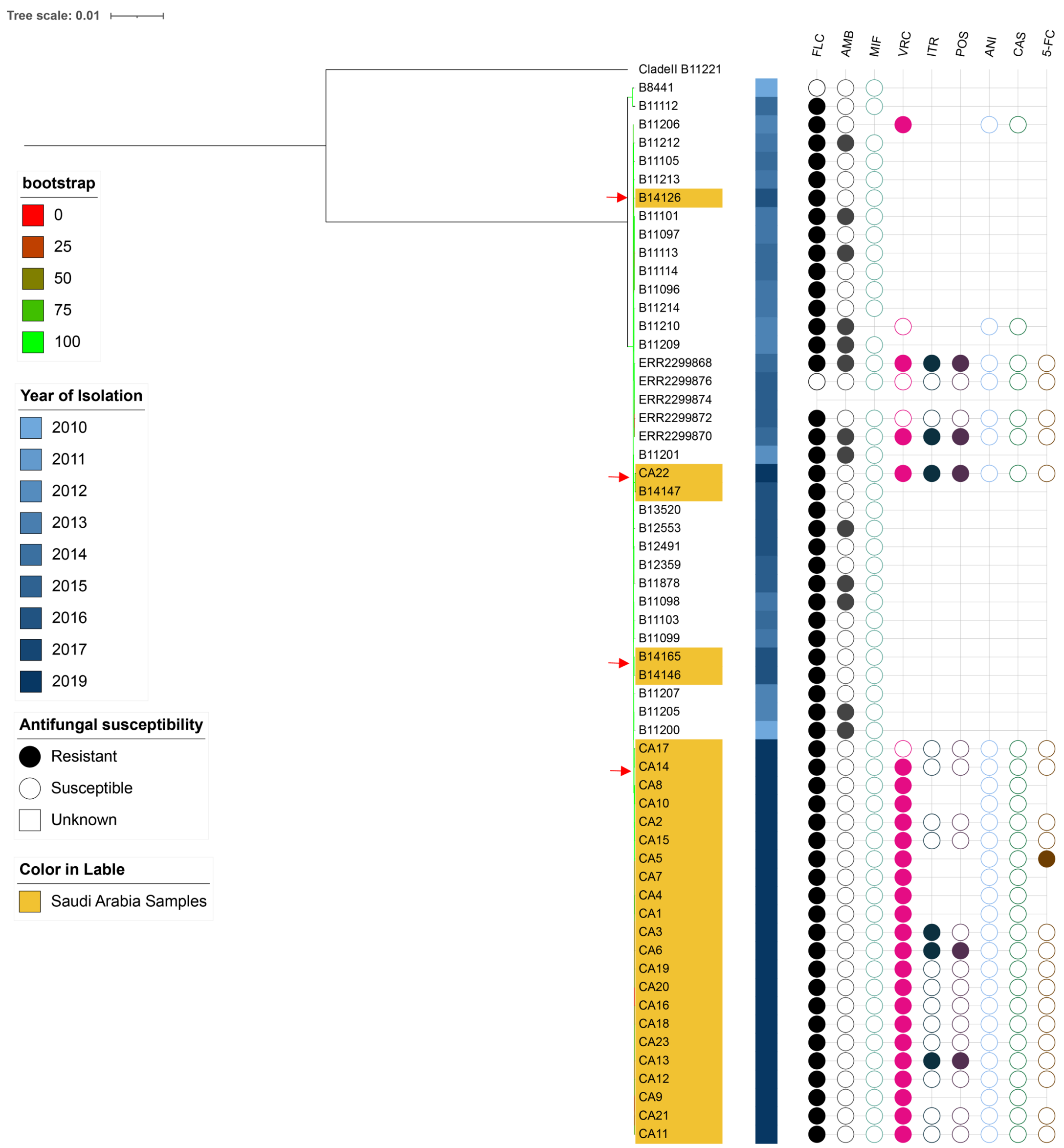

Supplementary Figure 2. Maximum-likelihood phylogenetic tree of the 58 Clade I *C. auris.* A Phylogenetic tree was based on SNP information and rooted in Clade II B11221 lineage as the outgroup. The Saudi Arabian strains were labelled with yellow colours and 4 independent introduction events (2017 and 2019) were indicated by red triangles. The samples derived from the same patients were indicated by the same colour of star symbol, the bootstrap values by the legend on the left. Information of the year of isolation was indicated by the legend on the left. The antifungal susceptibility profile is displayed on the right panel, showing the resistance (colorful circles), susceptibility (empty circles), or unknown status of each isolate to various antifungal drugs, including fluconazole (FLC), amphotericin B (AMB), voriconazole

(VRC), itraconazole (ITR), posaconazole (POS), anidulafungin (ANI), caspofungin (CAS), and 5-flucytosine (5-FC). The raw tree file was attached in Supplementary File1.

Supplementary File 1. Evolutionary tree for Fig.S2 in Newick format, generated using IQ-TREE with 1000 bootstrap replicates.

(B11096:0.0000032739,((B11097:0.0000032739,(((((((((((((B11098:0.0000062161,(B11099:0.0000003286,B11103:0.0000003286)94:0.0000003287)100:0.0000397577,((B11878:0.0000185931,B12359:0.0000154719)74:0.0000032739,B12491:0.0000458741)100:0.0000642322)46:0.0000003287,((B12553:0.0000978817,B13520:0.0000336423)100:0.0000215125,(B14147:0.0000978842,CA22:0.0001192978)100:0.0002631111)71:0.0000003287)0:0.0000003287,((((B11200:0.0000032739,B11205:0.0000032739)100:0.0000244664,B11207:0.0000428162)95:0.0000091648,(B14146:0.0000185919,B14165:0.0000124318)100:0.0000672912)46:0.0000003287,(((CA10:0.0000003286,CA8:0.0000003286)100:0.0000672901,((((((((((((((CA11:0.0000124316,CA21:0.0000091653)64:0.0000032739,CA9:0.0000003286)36:0.0000003287,CA12:0.0000003286)31:0.0000003287,CA13:0.0000124315)61:0.0000003287,CA23:0.0000003286)43:0.0000003287,CA18:0.0000003286)66:0.0000003287,CA16:0.0000244657)66:0.0000032739,CA20:0.0000003286)50:0.0000003287,CA19:0.0000003286)14:0.0000003287,CA6:0.0000003286)54:0.0000003287,CA3:0.0000003286)48:0.0000003287,((CA1:0.0000003286,CA4:0.0000003286)100:0.0000305823,CA7:0.0000003286)1:0.0000003287)46:0.0000003287,CA5:0.0000003286)66:0.0000003287,(CA15:0.0000062160,CA2:0.0000003286)100:0.0000244656)51:0.0000003287)100:0.0000305827,(CA14:0.0000305832,CA17:0.0000825863)100:0.0000244655)100:0.0000672921)100:0.0000185922)100:0.0000244657,B11201:0.0000672922)100:0.0000642462,((B11112:0.0004621884,B8441:0.0002385690)100:0.0009583386,clade2_B11221:0.1151911032)100:0.0010880722)85:0.0000032739,B11206:0.0000003286)100:0.0001101140,B11212:0.0000672903)57:0.0000003287,B11105:0.0000917617)87:0.0000062202,B11213:0.0000703493)62:0.0000003287,B14126:0.0000550556)99:0.0000124305,(((B11209:0.0000003286,B11210:0.0000003286)29:0.0000003287,B11214:0.0000003286)87:0.0000062200,(ERR2299868:0.0000003286,(((ERR2299870:0.0000153630,ERR2299872:0.0000003286)60:0.0000032739,ERR2299874:0.0000091653)27:0.0000003287,ERR2299876:0.0000153631)88:0.0000062203)100:0.0000397559)100:0.0000183301)100:0.0000703500,B11101:0.0000003286)64:0.0000032739)84:0.0000003287,B11113:0.0000003286)80:0.0000003287,B11114:0.0000032739)

Reference:

1. Nascimento M, Sousa A, Ramirez M, Francisco AP, Carriço JA, Vaz C. PHYLOViZ 2.0: Providing scalable data integration and visualization for multiple phylogenetic inference methods. Bioinformatics **2017**;
2. Sahl JW, Lemmer D, Travis J, et al. NASP: an accurate, rapid method for the identification of SNPs in WGS datasets that supports flexible input and output formats. Microb genomics **2016**;
